# Unleashing patient voices: empowering adverse event assessment with complete patient-reported outcomes

**DOI:** 10.1093/oncolo/oyae105

**Published:** 2024-05-24

**Authors:** Thi Hanh Phung, Natalie Bradford, Erin Pitt, Kimberly Alexander

**Affiliations:** School of Nursing, Faculty of Health, N block, Queensland University of Technology, Kelvin Grove, 4059, Australia; Faculty of Nursing and Midwifery, Hanoi Medical University, Hanoi, Vietnam; School of Nursing, Faculty of Health, N block, Queensland University of Technology, Kelvin Grove, 4059, Australia; Cancer and Palliative Care Outcomes Centre, Centre for Healthcare Transformation, Queensland University of Technology, X Block, 66 Musk Avenue, Kelvin Grove, 4059, Australia; Cancer Nurses Society of Australia, 165 Sovereign Hill Drive, Gabbadah Western Australia, 6041, Australia; Centre for Children’s Health Research, Children’s Health Queensland Hospital and Health Services, South Brisbane, 4101, Australia; School of Nursing, Faculty of Health, N block, Queensland University of Technology, Kelvin Grove, 4059, Australia; Cancer and Palliative Care Outcomes Centre, Centre for Healthcare Transformation, Queensland University of Technology, X Block, 66 Musk Avenue, Kelvin Grove, 4059, Australia; School of Nursing, Faculty of Health, N block, Queensland University of Technology, Kelvin Grove, 4059, Australia; Cancer and Palliative Care Outcomes Centre, Centre for Healthcare Transformation, Queensland University of Technology, X Block, 66 Musk Avenue, Kelvin Grove, 4059, Australia; Cancer Nurses Society of Australia, 165 Sovereign Hill Drive, Gabbadah Western Australia, 6041, Australia; Centre for Children’s Health Research, Children’s Health Queensland Hospital and Health Services, South Brisbane, 4101, Australia

**Keywords:** Cancer, chemotherapy, feasibility study, patient-reported outcomes, PRO-CTCAE

## Abstract

The 124-item patient reported-outcome common terminology criteria for adverse events (PRO-CTCAE) questionnaire, assessing 78 symptoms, is widely used in cancer clinical trials to identify side effects. However, its regular use in routine cancer care is rarely reported. We aimed to investigate the feasibility of weekly PRO-CTCAE completion over 9 weeks in a prospective study with 30 patients with cancer undergoing chemotherapy. Participants were asked to complete electronic surveys with reminders, but no feedback or incentives. Only 136 (50%) of the planned 270 time points at which a PRO-CTCAE self-report was expected were completed, with an additional 21 (8%) partially completed, and represents a failure to achieve the expected level of completion. Patients reported experiencing up to 51 and a median of 30 symptoms across all time points, highlighting the complexity of symptom assessment in acute cancer care. While weekly implementation of the PRO-CTCAE may not be feasible outside of clinical trial settings, this study highlights the breadth of symptoms experienced.

## Teaser

This study investigated the feasibility of weekly PRO-CTCAE completion over 9 weeks in a prospective study with 30 patients with cancer undergoing chemotherapy.

## Introduction

Patient self-reported outcome measurements (PROMs), referring to measurements of health status that come directly from patients, have gained increasing recognition for timely management of chemotherapy-related symptoms.^1^ PROMs offer valuable insights, leading to improved management of symptoms, as well as increased patient satisfaction and stronger therapeutic relationships.^2^

The PRO-CTCAE is a widely used PROM with 124 items screening for 78 symptoms and its implementation holds great potential for both patients and clinicians. Patients’ ability to assess symptoms accurately is invaluable. As such, using the PRO-CTCAE enhances assessment and management of adverse events, resulting in improved symptom control, increased patient satisfaction, and stronger therapeutic relationships.

However, despite its widespread use in cancer clinical trials, the feasibility of integrating the complete PRO-CTCAE within routine clinical practice remains unexplored. Previous studies have often utilised only a subset of the 78-symptom library, and primarily focused on reporting symptoms within cancer clinical trials.^2-8^

The integration of PROMs in everyday cancer care encounters various obstacles, at many levels, including staff (lack of knowledge or time constraints), patients (language or technology barriers), and infrastructure (inappropriate technology platforms or technical problems).^9^ While such problems may well be managed in a clinical trial setting, these are difficult to control in real clinical situations.

In this study, our aim was to assess the feasibility implementing the PRO-CTCAE in non-research clinical setting to inform its potential integration in a prospective cohort study and in healthcare services for routine reporting of chemotherapy-related symptoms. Our purpose was to determine the viability of using the PRO-CTCAE to its full extent in routine care.

## Methods

In this prospective longitudinal pilot study, 30 patients from St Vincent’s Private Hospital Northside were consecutively recruited at the beginning of their chemotherapy cycle. Eligible patients were (1) aged over 18 years old, (2) undergoing anti-cancer treatment with at least 3 cycles of anti-cancer treatment remaining, (3) competent to give informed consent, and (4) had sufficient English to read and understand the information and consent materials.

Patients were asked to complete the PRO-CTCAE online through Research Electronic Data Capture, REDCap—an electronic data capture platform—in their homes weekly for 9 weeks across their chemotherapy cycles. A weekly email was sent to participants to complete the questionnaire with up to two reminders to encourage completion. Demographic and clinical variables were summarized and are described with mean and standard deviation (SD) if normally distributed or as median and range otherwise for continuous variables and as number (percentage) for categorical variables.

All statistical analyses were undertaken using SPSS V26.0 (IBM Corp, Armonk, New York, USA). The study received ethics approval (Reference number: 1600001208).

## Results

Over 4 months, 31 consecutive patients were screened to identify their eligibility against inclusion and exclusion criteria and invited to participate. Thirty patients agreed to participate. The median age of participants was 60 years, and 23 respondents (76.7%) were female. Breast cancer was the most common diagnosis, with 15 respondents (50%). The full list of demographic and clinical characteristics measured can be seen in Table 1. In the study, participants reported on 78 symptoms using the complete PRO-CTCTAE questionnaire. The median number of symptoms reported was 30 with 51 being the highest number of symptoms reported on any given survey, whilst some reported no symptoms at all.

**Table 1:** Patient characteristics.

Characteristics		*N* (%)
Age (years) Median (range)		60 (26-79)
Gender	Male	7 (23.3)
	Female	23 (76.7)
Diagnosis	Breast cancer	15 (50.0)
	Colorectal cancer	5 (16.7)
	Lung cancer	2 (6.7)
	Others	8 (26.6)
Marital status	Never married/partnered	2 (6.7)
	Married/partnered	24 (80)
	Divorced/separated	4 (13.3)
The highest education level	Secondary	11 (36.7)
	Certificate/diploma	10 (33.3)
	Bachelor or post-graduate	9 (30)
Living status	Live with partner/spouse/family/friends	26 (86.7)
	Live alone	4 (13.3)
Months since cancer diagnosis, Median (range)		3 (1-76)
Employment	Employed/self-employed	15 (50.0)
	Unemployed	5 (16.7)
	Retired	10 (33.3)
Ethnicity	British/Scottish/Welsh/Irish	15 (50)
	Southern Europe	2 (6.7)
	Central/Eastern Europe	2 (6.7)
	Northern/Western Europe	1 (3.3)
	Middle Eastern	1 (3.3)
	Other	8 (26.7)
Total household income (AUD)	<$70.000	15 (51.7)
	≥$70.000	14 (48.3)
BMI	Healthy weight (18.5 to 25)	8 (26.7)
	Overweight or obese (> 25)	22 (73.3)
Lost weight without trying	No	17 (56.7)
	Yes or unsure	13 (43.4)
Current level of exercise	No exercise	6 (20.0)
	Limited exercise	16 (53.3)
	Regular exercise	8 (26.7)
Smoking status	Former smoker	16 (53.3)
	Never smoker	14 (46.7)

Abbreviation: BMI, body mass index.

Table 2 shows the adherence rate by patients for PRO-CTCAE completion. Among the 30 patients enrolled in the study, 11 completed or partially completed all 9 surveys, and one patient completed 8 surveys.

**Table 2.** Patient adherence during 9 excessive weeks.

Patients	Week 1	Week 2	Week 3	Week 4	Week 5	Week 6	Week 7	Week 8	Week 9
1									
2									
3									
4									
5									
6									
7									
8									
9									
10									
11									
12									
13									
14									
15									
16									
17									
18									
19									
20									
21									
22									
23									
24									
25									
26									
27									
28									
29									
30									

Green color indicates completion, yellow color indicates partially completion, and red color indicates non-completion.

Out of the 270 expected PRO-CTCAE self-reports, 136 (50%) were fully completed, 21 (8%) were partially completed, and 113 (42%) were not completed.

## Discussion

To our knowledge, this is the first study to investigate the feasibility of repeatedly completing the full version of PRO-CTCAE in nine consecutive weeks in a clinical setting without assistance. The completion rate over the total study duration was 58% which is notably lower compared with previous studies that report PRO-CTCAE completion rates ranging from approximately 75% to 97%.^2-8^ There are 3 possible explanations for the low adherence in our study.

Firstly, problems related to the time frame for the completion of PRO-CTCAE over 9 weeks could be a barrier. The PRO-CTCAE questionnaire itself includes 124 questions for patients to respond to 78 different symptoms. This sheer volume of questions may have overwhelmed participants and acted as a deterrent to consistent completion. Moreover, the frequent data collection over the 9-week period could have been perceived as burdensome by the patients, further impacting their willingness to complete the surveys diligently. This, in turn, could have led to the lower completion rate observed in this study.

In a previous study, despite using the full version of PRO-CTCAE, participants only completed the tool at 3-time points (baseline, midcycle 1, and midcycle 2) which resulted in a high total adherence rate of 91%.^8^ Inclusion of fewer symptoms (ie, a subset of the whole PRO-CTCAE) may also contribute to a higher completion rate. For example, a 90% completion rate was reported when utilising 12 to 22 symptoms from the full PRO-CTCAE questionnaire.^2,3,5^ However, careful consideration is necessary to ensure comprehensive symptoms assessment, as noted in this study, participants reported up to 51 different symptoms and a median of 30 symptoms on any given survey.

Another possible reason for the low completion rate could be the mode of completion. Technical issues with electronic devices are reported as one of the main reasons for missing data in previous studies using electronic PROMs.^2-4,7^ We also previously identified that patients have different preferences for communicating about symptoms post-hospital discharge which included preference for modes including telephone, in-person, emails, and text messages.^10^ In this study, patients could only complete the PRO-CTCAE questionnaire online through REDCap at home with no technical support.

Lastly, the lack of incentives for completion could result in a lower adherence rate. In active clinical trials, health professionals follow patients up to encourage the completion of PRO-CTCAE.^2,3,5,6^ For example, coordinators directly called those who did not complete the survey to offer assistance.^2^ In another trial, patients’ PRO-CTCAE responses were reviewed by oncologists in their weekly clinic meetings; this may also encourage them to complete their responses.^3^ The absence of such an intervention in the current pragmatic study where participants completed the survey independently at home, with only email reminders could have contributed to the lower adherence rate.

## Conclusion

In summary, the unassisted completion rate of the entire PRO-CTCAE questionnaire was found to be low. Implementation of the complete questionnaire is infeasible in practice without human resourcing to support its implementation and ongoing use. This study highlights the importance of securing funding for positions that facilitate PROM completion both in research and in practice. Given the growing pressure to utilize technology for healthcare efficiency, these findings hold particular relevance in the current healthcare landscape.

## Data Availability

The data underlying this article will be shared on reasonable request to the corresponding author and with appropriate ethical approval.
